# Heart Transplantation in Congenital Heart Disease

**DOI:** 10.2174/157340311797484268

**Published:** 2011-05

**Authors:** Francesco Parisi

Orthotopic heart transplantation has been performed in pediatric patients for 27 years. Indications changed through the years, showing the increase of patients receiving a heart transplant for congenital heart defects, after having experienced repeated surgical palliations.

At present, 1-year survival > 65% can be expected following pediatric heart transplantation. Morbidity other than hypertension is uncommon and catch-up growth and hemodynamic rehabilitation to normal childhood functional status is the likely outcome. The quality of life can be normal. The diagnosis of CHD is a risk factor for mortality in OHT recipients based on the ISHLT data. For mortality *within one year* from transplant, the relative risk for pediatric OHT patients with CHD is 2.17 for pediatric OHT recipients older than one year. For *5-year mortality*, the hazard ratio for CHD patients vs. others has been reported as 1.50. With regards to long term survival data, the ISHLT data reveal improvements over time for all pediatric OHT recipients. An important factor for success in CHD patients is careful patient evaluation prior to OHT. Guidelines have been developed to assist in the assessment of the risk profile of this very heterogeneous patient group. Broadly, both biological and psycho-social factors have to be taken into consideration. 

Heart transplantation remains the only hope for children with some forms of complex congenital heart disease and some infants and children with failed surgical intervention. In specific cases, OHT may offer a better likelihood of long terms survival than conventional medical and surgical therapy although the shortage of donor organs limits the feasibility of the primary OHT approach. Progress in immunology has enhanced the success rate of long term management of graft rejection. The overall survival after OHT for congenital heart disease has improved compared to all other indications. There is an increasing body of experience with the specific challenges of OHT for congenital heart disease, both as primary treatment option or as rescue after other therapies have failed.

These indications to transplant for congenital heart defects, the selection criteria of patients, the related risks and possible complications are still not well defined.

For this reason, we propose a text which deals with both the problems of patients suffering from congenital heart disease who are referred to heart transplant programs and the possible future prospects.

The review discusses the indications for heart transplantation, various phases of the transplant process, the immunosuppressive treatment and the role of heart transplant in the group of congenital patients. 

